# Role of inflammation in determining the severity of COVID-19 infection in patients with diabetes: A comparative study

**DOI:** 10.1097/MD.0000000000036641

**Published:** 2023-12-15

**Authors:** Besher A. Gharaibeh, Sawsan Abuhammad, Obieda Haneyah, Amat Al-Khaleq O. Mehrass

**Affiliations:** a Department of Adult Health, Faculty of Nursing, Jordan University of Science and Technology, Irbid, Jordan; b Department of Maternal and Child Health, Faculty of Nursing, Jordan University of Science and Technology, Irbid, Jordan; c Obstetric and Gynecology Department, Faculty of Medicine, Thamar University, Dhamar, Yemen

**Keywords:** COVID, 19, 19, COVID, 19 severe, diabetes, inflammatory process

## Abstract

There is a need to consider the geographical origins when studying the association between COVID-19 and the comorbid conditions. To examine the role of inflammation in determining the severity of COVID-19 among hospitalized patients with diabetes and compare these roles with those who does not have diabetes. A cross sectional comparative design was used with a convenience sample of 352 patients. Samples were collected from hospitalized patients with COVID-19 who were divided into 2 groups (diabetes and non-diabetes). Data regarding results of selected inflammatory markers and sociodemographic were collected. The severity of COVID-19 differed significantly between the diabetes and non-diabetes groups (Chi square = 25.58 *P* < .05). There was significant difference in the mean scores of neutrophil counts, monocyte count, Basophil count, erythrocyte sedimentation rate, partial thromboplastin time, C-creative protein, platelets, white blood cells, and mean cellular hemoglobin center between those with and those without diabetes. The diabetes were shown more increased in the predictors and severity of the COVID-19 disease. However, neutrophil to lymphocyte ratio, neutrophil count, and age were the significant predictors of the severity level of COVID-19 among patients with diabetes. In conclusion, our study addressed the influence of having diabetes among hospitalized patients with moderate and severe COVID-19 infection. The results showed that severity of COVID-19 infection was affected by diabetes where those with diabetes had more tendency to suffer from the severe form of the disease rather that the moderate level.

## 1. Introduction

Coronavirus disease (COVID-19-19) is an infectious disease caused by the SARS-CoV-2 virus that targets the respiratory system causing various symptoms that include and can lead to death.^[1–3]^ COVID-19 has affected over 100 million people worldwide and caused over 6 million deaths so far, and over 700 thousand confirmed new cases are reported daily.^[4]^ In Jordan, the total number of cases so far exceeded 1.7 million and the fatalities so far had surpassed 14 thousand. The report on admission showed that about 159 cases are admitted per week.^[5]^

Diabetes had been addressed as a main factor that increases risk of complications and mortality for those with COVID-19 infection.^[6–8]^ The relationship between diabetes and COVID-19 infection was discussed by Gangadaran et al^[9]^ in their review paper where they indicated that diabetes is linked^[10]^ to the severity of the COVID-19 and its rapid progression. The negative impact of diabetes on the health outcomes of patients with COVID-19 was attributed to hyperglycemic changes and immune responses. In their article, the authors argued that hyperglycemia leads to metabolic changes and significant increase in the monocytes. These processes can upregulate the proteins involved in cell damage and stimulate several immune cells which causes elevation in some inflammatory cytokines such as tumor necrosis factor α (TNF-α), interleukin 1 beta, and interleukin 6 proteins (IL-6). Although more knowledge about the relationship between diabetes and COVID-19 is emerging, challenges in understanding the interrelationship between COVID-19 and diabetes remain because this relationship is complex.^[10–12]^

Varikasuvu et al^[13]^ conducted a systematic review study to analyze and evaluate studies in the literature that examined the changes in inflammatory and coagulation markers in patients with diabetes. In their review paper the authors stated that despite the plentiful of studies addressed inflammation in patients with diabetes and COVID-19-19, much attention must be focused on comparing diabetes versus non-diabetes COVID-19 patients, and further studies are needed in future to explain the relationships between inflammation, diabetes, and COVID-19 infection. Moreover, Bradley et al^[14]^ in their meta-analysis study reported that despite the overwhelming number of studies that concluded effect of diabetes on COVID-19 mortality, there is evidence that showed this effect is mediated by different factors and the difference in COVID-19 health outcomes was not different between diabetes and non-diabetes patients in some cases. Also, the authors stated that there were inconsistencies and variations in the definitions of the outcomes addressed in the reviewed studies, thus, the results of the many studies should be interpreted in their study populations. So, studies should consider diverse cohorts specifically individuals hospitalized for COVID-19 infection.^[15]^ Moreover, when addressing the role of diabetes in COVID-19 infection, there is a need to factor in the geographical origins because there is variability across the world regions which may significantly skew overall trends.^[16]^ In addition to health-related problems, the pandemic has also become the interest in the research studies, several studies have shown increased pro-inflammatory cytokines in serum of COVID-19 patients. However, the role of inflammatory markers among diabetic patients and none in monitoring the severity of COVID-19 is still unclear.^[17]^

The purpose of our study is to examine the role of inflammation in determining the severity of COVID-19 among hospitalized patients with diabetes. Studying these relationships can provide better scientific knowledge about the mechanisms by which the immune system and the inflammatory process are affected during COVID-19 infection, which may assist in generating strategies and protocols that can help in treating the infection and improving the health outcomes for patients with COVID-19-19.

Research questions are:

- Are there differences in the level of COVID-19 severity between diabetes and non-diabetes groups?
- Are there differences in the mean scores of the inflammatory markers between diabetes and non-diabetes groups?
- Are there differences in the mean scores of the inflammatory markers between the 2 level of COVID-19 severity (moderate and severe) groups?
- What are the inflammatory markers that significantly influence the level of COVID-19 severity among diabetes and no-diabetes groups?

## 2. Methods

### 2.1. Design

A cross-sectional design was used to address the severity of COVID-19 in patients with and patients without diabetes. The patient record was accessed and used to determine whether the patient has or does not have diabetes based on the medical diagnosis.

### 2.2. Sample

A convenience sampling technique was used to recruit participants from the targeted hospitals. The inclusion criteria were: (1) patient have confirmed diagnosis of moderate or severe COVID-19-19; (2) equal or more than 18 years of age; and (3) can speak and read Arabic language. The exclusion criteria were those diagnosed as critical cases that related to respiratory or cardiac system or intubated patients. Also, people with chronic diseases related to cardiac system were excluded. The Jordanian Ministry of Health protocol (2022) stated that the confirmed COVID-19 case is defined as the case that is laboratory-confirmed by a PCR examination through a positive result to detect the SARS-CoV2 virus.

The protocol stated that cases are diagnosed in Jordan only by adopting the polymerase chain reaction test. Test samples are taken using nasopharyngeal swab, sputum sample, or pulmonary lysing sample. Two or more samples can be taken depending on the availability of the samples and according to the opinion of the attending physician, as required by the patient’s condition.

The COVID-19 infection in Jordan is diagnosed based on the results of Real-Time PCR (RT-PCR) test (COVID-19 MDx RT-PCR COVID-19 DETECTION KITS). Those tests are FDA approved by Jordanian Food and Drug Administration. The RT-PCR is an in vitro diagnostic that is used to detect SARS-CoV-2 in nasopharyngeal/oropharyngeal swabs, anterior/mid-turbinate nasal swabs. The estimated sensitivity of the test was reported around 80% and specificity about 99%.^[18]^

The minimum sample size was estimated using G*Power 3.1 for ANOVA test. The following parameters were used to estimate the sample size: alpha = .05, beta = .80, and effect size of 0.07 (representing small to medium effect size). An estimated dropout percentage of 15% was considered. Thus, the minimum final estimated sample size was 350 participants. The final recruited sample was 352 participants. These participants were all their biomarkers test were done with hospitals. The sample size was calculated to determine the required number to answer the questions of the paper.

### 2.3. Sociodemographic and potential confounders

A sociodemographic sheet was used to collect data regarding various demographic characteristics such as age, gender, marital status, and other characteristics. Also, questions to collect data regarding potential confounders such as comorbid conditions and receiving COVID-19 vaccine was included in this sheet. These data were confirmed by returning to the patients’ charts.

### 2.4. Severity of COVID-19 infection

The severity of COVID-19 infection was measured as an ordinal variable to determine whether the patient is considered to have moderate or severe COVID-19 19 infections. The protocol that was proposed by the diagnostic and treatment protocol for patients with the emerging coronavirus (COVID-19-19) issued by the Jordanian Ministry of Health and approved by the National Committee for Epidemic Control; was used to describe if the case is considered moderate or severe COVID-19 infection as previously mentioned. Jordan protocol indicates that the patient who suffers from symptoms and signs of lower respiratory tract infections (bronchitis or pneumonia), including shortness of breath, and the percentage of hemoglobin saturation with oxygen is more than 94% is considered as a moderate COVID-19 infection. On the other hand, the severe case is a patient who suffers from pulmonary infections with shortness of breath, and the percentage of hemoglobin saturation with oxygen is <94%.However, the X-rays and CT scans were considered to improve the severity of the cases.

### 2.5. Inflammatory biomarkers

The results of the inflammatory biomarkers for the corresponding participants were gathered from the hospital records. The protocol by the MOH described the required tests for all admitted/hospitalized patients with COVID-19 19 infection; this protocol indicated that the following tests should be done for the hospitalized COVID-19 19 patients on daily basis.

Inflammatory and coagulation markers tested according to the management protocol were neutrophil count, lymphocyte count, monocyte count, eosinophil count, basophil count, D-dimer, creative reactive protein (CRP), procalcitonin (PCT), IL-6, erythrocyte sedimentation rate (ESR), mean cellular volume, mean corpuscular hemoglobin concentration (MCHC), mean corpuscular hemoglobin, platelets, brain natriuretic peptide, partial thrombin, partial thrombin time (PTT), International normalized ratio, ferritin, and lactate dehydrogenase. Moreover, the neutrophil to lymphocyte ratio (NLR) was calculated by dividing the neutrophil absolute count by the lymphocyte absolute count.

### 2.6. Settings

Three of the hospitals that were allocated by the Jordanian government as hospitals to receive patients with COVID-19 19 infection were selected for data collection. Only certain hospitals were selected by the government to admit COVID-19 19 patients. Other hospitals were instructed to transfer any COVID-19 patients to those previously identified as precaution to control disease transmission.

### 2.7. Data collection procedure

Data collection was done between May 1st, 2022, and June 4th, 2022. After acquiring the IRB and the approval from the selected hospitals, the investigator introduced the topic to the head nurse and staff nurses to provide clarification regarding the study and the questionnaire. The investigator distributed the demographic questionnaire with other related questions to the potential participants who met the inclusion criteria that previously described. Those who agreed to participate signed an informed consent and completed the questionnaire that related demographic and other important information that listed before. The investigator addressed any concerns and questions from the potential participants and checked the questionnaire to ensure there was no missing data. If missing data were noticed, the participants were instructed to fill that part appropriately. Results of the inflammatory markers were collected from the corresponding participant’s records by the investigator and were recorded on printed tables.

### 2.8. Data analysis procedures

The SPSS program version 25 (IBM Corp, Armonk, RRID:SCR_016479) was used to analyze the data. The study variables were explained using descriptive statistics (e.g., frequency, percentage, mean, standard deviation, and range). Using the receiver operating characteristic (ROC) and area under the ROC curve (AUC) analysis, each marker was plotted against COVID severity with the value of state variable set as (2) indicating severe COVID infection. Logistic regression to determine the predictors, Chi square to determine the difference between DM and non-DM participants, and ANOVA tests was used to determine the difference between different degree of DM patients. *P*-value for our study was determined at level of (.05).

### 2.9. Ethical consideration

This study approved by Jordan University of Science and Technology (IRB number 656-2021) (Jordan University of Science and Technology). The authors were explained all the risks and benefits for the participants. All participants were signed consent form for participation in this study. The written informed consent were received from all the participants.

## 3. Results

### 3.1. Sociodemographic characteristics

A total of 352 patients participated in the study. Mean age was 57 years old (SD = 19 years). More than half of the participants were female (n = 200). Meanwhile, 63.1 percent of the sample reported having fatigue (n = 222). More than three-fourths of patients had severe COVID-19 (n = 274, 77.8%). The rest had moderate COVID-19 (n = 78, 22.2%). See Table 1 for demographic characteristics.

**Table 1:** sociodemographic characteristics of the study sample.

	Mean	SD
Age	57	19
	*Frequency (n*)	*Percentage (%*)
*Gender*		
Male	152	43.3
Female	200	56.7
*Marital status*		
Single	56	15
Married	216	62
Divorced	52	15
Widow	28	8
*Employment status*		
Unemployed	207	58.8
Employed	90	25.6
Retired	55	15.6
*Educational attainment*		
Did not go to school	44	12.5
Elementary	27	7.7
High school/secondary	69	19.7
Diploma	97	27.4
Bachelor’s degree/undergraduate	92	26.2
Postgraduate	23	6.6

### 3.2. Are there differences in the level of COVID-19 severity between diabetes and non-diabetes groups?

To test for presence of significant difference in these groups, chi square test was conducted. The results showed that the Pearson Chi square was (Chi square = 25.58 *P* < .05). The descriptive tables showed that a total of 274 patients had severe COVID-19 which 166 had diabetes and 108 did not have diabetes. Meanwhile, only 22 patients with diabetes had moderate COVID-19 whereas 56 patients with no diabetes had moderate COVID-19 infection.

**Figure 1.**
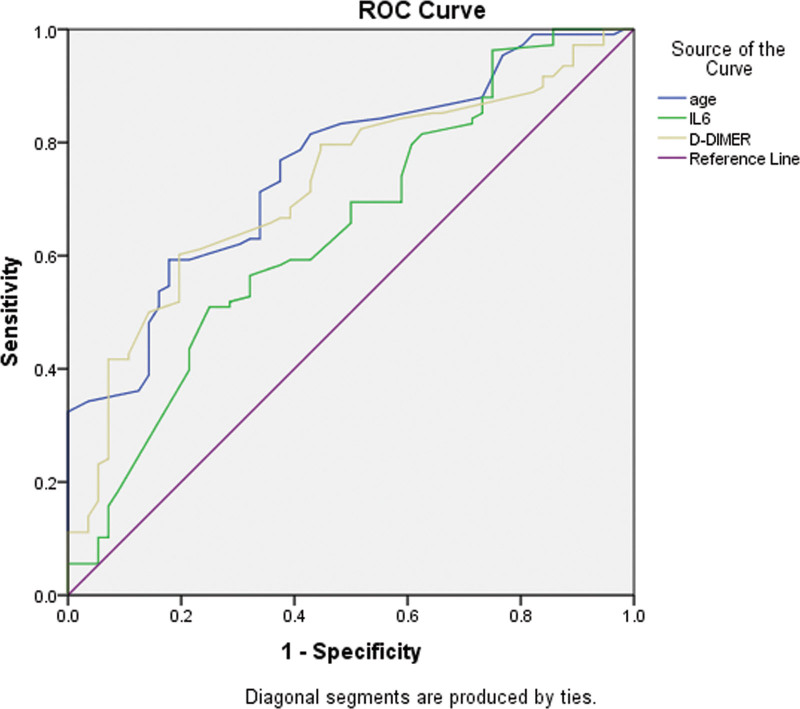
Receiver operating characteristic curve (ROC curve) for the main influencing factors (i.e., age, IL-6, and D-DIMER) model plotted against the severity of COVID infection in patients with no diabetes.

**Figure 2.**
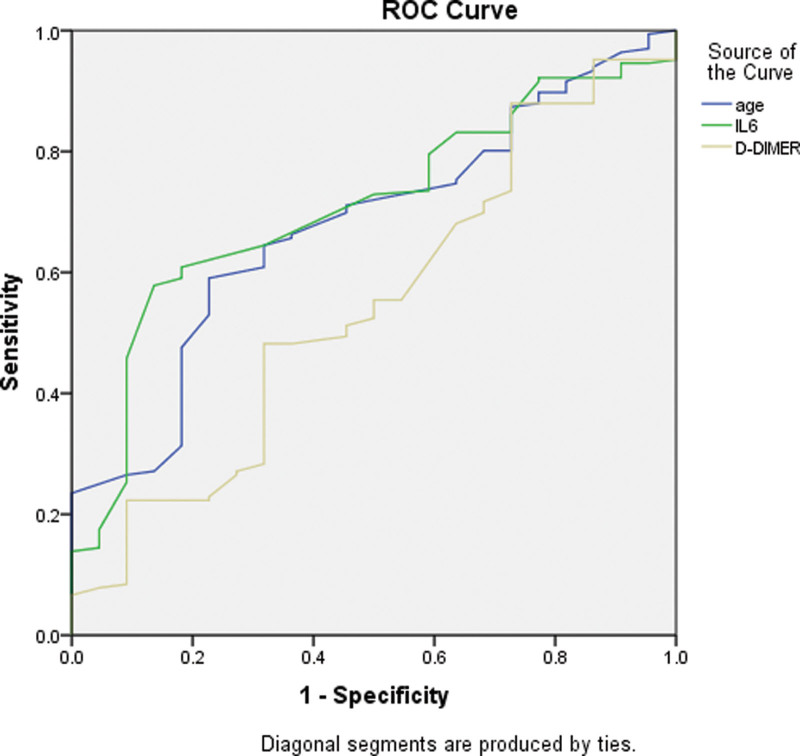
Receiver operating characteristic curve (ROC curve) for the main influencing factors (i.e., age, IL-6, and D-DIMER) model plotted against the severity of COVID infection in patients with diabetes.

Further analyses were conducted to test the relationship between the inflammatory markers and the severity of COVID-19 infection. These analyses were conducted to determine the accuracy of the individual classification models representing the relationship between the inflammatory marker of interest with the severity of COVID infection. Using the ROC and AUC analysis, each marker was plotted against COVID severity with the value of state variable set as (2) indicating severe COVID infection. For patients with no diabetes, the results of the analyses showed that most of the markers of interest had an AUC above the set level of (0.5). D-DIMER was the marker with the highest AUC among all the established models. Moreover, for patients with diabetes, the analyses showed thatIL-6 had the model with highest AUC. Age was also included in the analyses and was established as an important factor that contributed to the severity of COVID infection. See Figures 1 and 2 for the ROC curves in patients with diabetes, and patients with no diabetes. See Table 2 and 3.

**Table 2:** Area under the curve for the models representing the inflammatory markers to determine their effect on the severity of COVID-19 in patients with no diabetes.

Test variable	AUC
Age	.754
(DDIMER)	.721
(MCHB)	.678
(CRP)	.674
(BNB)	.674
(ESR)	.660
(PTT)	.657
(IL6)	.646
(LDH)	.643
(PT)	.636
(INR)	.608
(MCV)	.577
Eoseno_count	.577
(PCT)	.571
(FERRITIN)	.569
(REDCELLDW)	.545
Mono_count	.543
(PCV)	.524
Baso_count	.511
Lympho_count	.493
Neutro to lympho ratio	.487
Neutro_count	.436
(ERRYTHRO)	.434
(WBC)	.428
(PLATELET)	.348

BNP = brain natriuretic peptide, CRP = C-creative protein, ESR = erythrocyte sedimentation rate, INR = International Normalized Ratio, LDH = lactate dehydrogenase, MCH = mean corpuscular hemoglobin, PT = partial thrombin, PTT = partial thromboplastin time, WBC = white blood cells.

**Table 3:** Area under the curve for the models representing the inflammatory markers to determine their effect on the severity of COVID-19 in patients with diabetes.

Test result variable(s)	AUC
(IL6)	.708
Age	.676
(ESR)	.612
(PTT)	.611
(PCT)	.605
(CRP)	.592
(MCHB)	.576
(PT)	.568
(ERRYTHRO)	.566
(MCHBC)	.559
(INR)	.555
(BNP)	.549
(DDIMER)	.546
(FERRITIN)	.530
Neutro to lympho ratio	.524
(MCV)	.518
(WBC)	.512
(LDH)	.506
Mono_count	.481
(PCV)	.481
Baso_count	.474
(PLATELET)	.471
Eoseno_count	.445
Neutro_count	.436
(REDCELLDW)	.407
Lympho_count	.360

BNP = brain natriuretic peptide, CRP = C-creative protein, ESR = erythrocyte sedimentation rate, INR = international normalized ratio, LDH = lactate dehydrogenase, MCH = mean corpuscular hemoglobin, PT = partial thrombin, PTT = partial thromboplastin time, WBC = white blood cells.

### 3.3. Are there differences in the mean scores of the inflammatory markers between diabetes and non-diabetes groups?

To answer this research question, ANOVA test was conducted. The results showed that the mean scores of neutrophil counts, monocyte count, Basophil count, ESR, PTT, CRP, platelets, white blood cells, and MCHC differed significantly between those with and those without diabetes. See Table 4 for the ANOVA test with having or not having diabetes as grouping variable.

**Table 4:** ANOVA test results to assess for significant difference in the mean scores of the tested inflammatory markers between patients with and patients without diabetes.

		N	Mean	95% Confidence interval for mean		*P*-value
				Lower bound	Upper bound	
NLR	No	164	5.36	4.54	6.18	.312
	Yes	188	6.02	5.05	6.99	
	Total	352	5.71	5.07	6.35	
Neutrophil count	No	164	4716.17	4193.29	5239.06	.027
	Yes	188	5817.62	5028.85	6606.40	
	Total	352	5304.45	4816.62	5792.27	
Lymphocyte count	No	164	1611.13	1386.58	1835.67	.340
	Yes	188	1813.66	1475.40	2151.91	
	Total	352	1719.30	1511.22	1927.37	
Monocyte count	No	164	728.70	625.01	832.39	.021
	Yes	188	903.10	797.92	1008.28	
	Total	352	821.85	747.55	896.14	
Eosinophil count	No	164	154.64	117.95	191.33	.885
	Yes	188	150.44	107.65	193.22	
	Total	352	152.39	123.99	180.79	
Basophil count	No	164	3.19	0.96	5.42	.005
	Yes	188	11.18	6.35	16.01	
	Total	352	7.46	4.65	10.26	
(ESR)	No	164	27.80	24.71	30.90	.010
	Yes	188	33.60	30.51	36.70	
	Total	352	30.90	28.70	33.11	
(IL6)	No	164	32.98	25.39	40.56	.346
	Yes	188	38.16	30.51	45.81	
	Total	352	35.75	30.36	41.13	
(LDH)	No	164	293.50	248.32	338.69	.168
	Yes	188	259.33	236.45	282.21	
	Total	352	275.25	250.97	299.53	
(FERRITIN)	No	164	490.13	399.66	580.61	.324
	Yes	188	427.72	341.92	513.52	
	Total	352	456.80	394.75	518.84	
(PTT)	No	164	27.20	26.22	28.17	.014
	Yes	188	28.66	27.99	29.33	
	Total	352	27.98	27.40	28.56	
(BNB)	No	164	202.73	178.74	226.71	.075
	Yes	188	233.50	209.66	257.34	
	Total	352	219.16	202.23	236.10	
(CRP)	No	164	44.64	36.48	52.81	.000
	Yes	188	88.82	74.65	103.00	
	Total	352	68.24	59.49	76.98	
(PCT)	No	164	1.95	0.96	2.94	.777
	Yes	188	1.77	0.99	2.55	
	Total	352	1.85	1.24	2.47	
(DDIMER)	No	164	2.40	2.14	2.66	.033
	Yes	188	2.79	2.54	3.05	
	Total	352	2.61	2.43	2.79	
(PT)	No	164	15.26	14.64	15.87	.761
	Yes	188	15.37	14.94	15.80	
	Total	352	15.32	14.95	15.68	
(INR)	No	164	1.29	1.23	1.36	.233
	Yes	188	1.98	0.92	3.05	
	Total	352	1.66	1.09	2.23	
(PLATELET)	No	164	252.96	239.22	266.71	.006
	Yes	188	285.01	267.24	302.79	
	Total	352	270.08	258.56	281.60	
(WBC)	No	164	10.04	9.40	10.68	.002
	Yes	188	11.58	10.84	12.32	
	Total	352	10.86	10.36	11.36	
(MCV)	No	164	81.79	80.10	83.48	.099
	Yes	188	83.57	82.25	84.89	
	Total	352	82.74	81.68	83.80	
(MCHB)	No	164	29.18	28.56	29.81	.116
	Yes	188	29.83	29.30	30.36	
	Total	352	29.53	29.12	29.93	
(MCHBC)	No	164	32.25	31.74	32.76	.005
	Yes	188	31.19	30.67	31.72	
	Total	352	31.68	31.31	32.05	

BNP = brain natriuretic peptide, CRP = C-creative protein, ESR = erythrocyte sedimentation rate, LDH = lactate dehydrogenase, INR = International Normalized Ratio, MCH = mean corpuscular hemoglobin, NLR = neutrophil to lymphocyte ratio, PT = partial thrombin, PTT = partial thromboplastin time, WBC = white blood cells.

### 3.4. Are there differences in the mean scores of the inflammatory markers between the 2 level of COVID-19 severity (moderate and severe) groups

The mean scores of the monocyte count, BNB, CRP, D-Dimer, partial thrombin, PTT, IL-6, ESR, platelets, and mean corpuscular hemoglobin differed significantly between those with moderate COVID-19 severity and those with severe COVID-19 infection. See Table 5 to see the results of the ANOVA test with COVID-19 severity as grouping variable.

**Table 5:** logistic regression to determine factors that influence COVID severity among patients with and those without diabetes.

Diabetes mellitus			B	Sig.	95% C.I. for EXP(B)	
					Lower	Upper
Step 1	No	NLR	−.088	.329	0.77	1.09
		Neutrophil count	.000	.646	1.00	1.00
		Lymphocyte count	−.001	.028	1.00	1.00
		Monocyte count	.000	.808	1.00	1.00
		Eosinophil count	.001	.611	1.00	1.01
		Basophil count	.024	.254	0.98	1.07
		BNB	.007	.028	1.00	1.01
		CRP	.004	.494	0.99	1.02
		PCT	−.110	.015	0.82	0.98
		DDIMER	.355	.192	0.84	2.43
		PT	.003	.987	0.71	1.41
		INR	−1.005	.429	0.03	4.41
		PTT	−.007	.919	0.87	1.14
		FERRITIN	−.002	.022	1.00	1.00
		LDH	.004	.065	1.00	1.01
		IL6	.010	.253	0.99	1.03
		ESR	.002	.911	0.97	1.03
		PLATELET	−.004	.252	0.99	1.00
		WBC	−.134	.099	0.75	1.03
		MCV	−.010	.694	0.94	1.04
		MCHB	.111	.337	0.89	1.40
		MCHBC	−.041	.696	0.78	1.18
		Age	.094	.001	1.04	1.16
		GENDER	−1.730	.014	0.05	0.70
		Heart Diseases	.600	.519	0.29	11.31
		HTN	−.913	.322	0.07	2.45
		KIDNEY Disease	−1.782	.079	0.02	1.23
		VACCINE	1.749	.035	1.13	29.34
		Constant	−.520	.930		
Step 2	Yes	NLR	.190	.049	1.00	1.46
		Neutrophil count	−.001	.009	1.00	1.00
		Lymphocyte count	.000	.230	1.00	1.00
		Monocyte count	−.001	.456	1.00	1.00
		Eosinophil count	.001	.592	1.00	1.01
		Basophil count	−.010	.626	0.95	1.03
		BNB	.005	.133	1.00	1.01
		CRP	.012	.144	1.00	1.03
		PCT	1.608	.187	0.46	54.48
		DDIMER	−.471	.084	0.37	1.07
		PT	−.162	.264	0.64	1.13
		INR	−.125	.055	0.78	1.00
		PTT	.223	.132	0.94	1.67
		FERRITIN	−.001	.247	1.00	1.00
		LDH	−.002	.264	0.99	1.00
		IL6	.030	.134	0.99	1.07
		ESR	.038	.171	0.98	1.10
		PLATELET	.001	.737	0.99	1.01
		WBC	.347	.085	0.95	2.10
		MCV	.065	.175	0.97	1.17
		MCHB	−.047	.689	0.76	1.20
		MCHBC	.287	.071	0.98	1.82
		Age	.083	.025	1.01	1.17
		GENDER	−.378	.700	0.10	4.69
		Heart Diseases	1.557	.088	0.79	28.37
		HTN	−1.361	.191	0.03	1.97
		KIDNEY Disease	−.473	.546	0.13	2.89
		VACCINE	.354	.760	0.15	13.79
		Constant	−21.354	.031		

BNP = brain natriuretic peptide, CRP = C-creative protein, ESR = erythrocyte sedimentation rate, INR = International Normalized Ratio, LDH = lactate dehydrogenase, MCH = mean corpuscular hemoglobin, NLR = neutrophil to lymphocyte ratio, PT = partial thrombin, PTT = partial thromboplastin time, WBC = white blood cells.

### 3.5. What are the inflammatory markers that significantly influence the level of COVID-19 severity among diabetes and no-diabetes groups?

To answer this research question, 2 logistic regression tests were conducted. In these 2 logistic tests, the same markers and variables were entered to the equations. First, binary logistic regression to determine the inflammatory markers that significantly predicted the likelihood of the patient to have moderate or severe COVID-19 infection among patients with no diabetes. The results of the analysis showed that (for patients with no diabetes) lymphocyte count, BNB, PCT, age, gender, and whether received COVID-19 vaccine. The test demonstrated the significance of the model (with no diabetes) (model chi square = 77.42; df = 23; *P* < .05) and Nagelkerke R squared = 0.52 indicating that about half the variability of the severity of COVID-19 was predicted/explained by the model.

Then, a second logistic regression was done to assess the relationship between the inflammatory markers and the severity of COVID-19 in patients with diabetes. This analysis showed that a different set of inflammatory markers were significantly associated with the severity of COVID-19 in patients with diabetes. These inflammatory markers included NLR, neutrophil count (absolute count), and age. The test demonstrated the significance of the model (with diabetes) (model chi square = 52.4; df = 23; *P* < .05) and Nagelkerke R squared = 0.47 indicating that approximately half the variability of the severity of COVID-19 was predicted/explained by the model. See Table 6 for the variables that predicted the severity and the corresponding odd ratios.

**Table 6:** ANOVA test results to assess for significant difference in the mean scores of the tested inflammatory markers between patients with moderate and severe COVID-19 infection.

		N	Mean	95% confidence interval for mean		*P*-value
				Lower bound	Upper bound	
NLR	Moderate	78	5.39	4.05	6.74	.607
	Severe	274	5.80	5.07	6.54	
	Total	352	5.71	5.07	6.35	
Neutrophil count	Moderate	78	5377.03	4506.66	6247.40	.876
	Severe	274	5283.78	4705.55	5862.02	
	Total	352	5304.44	4816.62	5792.27	
Lymphocyte count	Moderate	78	1923.25	1511.72	2334.79	.304
	Severe	274	1661.23	1420.11	1902.36	
	Total	352	1719.29	1511.22	1927.37	
Monocyte count	Moderate	78	662.04	572.10	751.99	.024
	Severe	274	867.33	775.86	958.81	
	Total	352	821.84	747.55	896.14	
Eosinophil count	Moderate	78	99.93	75.63	124.25	.052
	Severe	274	167.32	131.63	203.02	
	Total	352	152.39	123.99	180.79	
Basophil count	Moderate	78	6.80	1.38	12.23	.808
	Severe	274	7.64	4.37	10.91	
	Total	352	7.45	4.65	10.26	
(ESR)	Moderate	78	156.26	130.08	182.45	.000
	Severe	274	237.06	217.06	257.07	
	Total	352	219.16	202.23	236.10	
(IL6)	Moderate	78	40.40	29.04	51.78	.001
	Severe	274	76.16	65.56	86.76	
	Total	352	68.23	59.49	76.98	
(LDH)	Moderate	78	1.80	0.24	3.37	.931
	Severe	274	1.86	1.21	2.53	
	Total	352	1.85	1.24	2.47	
(FERRITIN)	Moderate	78	1.88	1.59	2.18	.000
	Severe	274	2.81	2.60	3.03	
	Total	352	2.60	2.43	2.79	
(PTT)	Moderate	78	14.62	14.08	15.16	.045
	Severe	274	15.51	15.08	15.96	
	Total	352	15.31	14.95	15.68	
(BNB)	Moderate	78	2.51	-0.05	5.09	.114
	Severe	274	1.41	1.31	1.53	
	Total	352	1.66	1.09	2.23	
(CRP)	Moderate	78	26.18	24.82	27.54	.001
	Severe	274	28.49	27.86	29.12	
	Total	352	27.97	27.40	28.56	
(PCT)	Moderate	78	420.76	338.00	503.52	.543
	Severe	274	467.05	390.72	543.39	
	Total	352	456.79	394.75	518.84	
(DDIMER)	Moderate	78	249.34	212.93	285.77	.263
	Severe	274	282.62	253.16	312.09	
	Total	352	275.25	250.97	299.53	
(PT)	Moderate	78	21.79	16.61	26.99	.006
	Severe	274	39.71	33.02	46.41	
	Total	352	35.74	30.36	41.13	
(INR)	Moderate	78	24.06	19.57	28.57	.001
	Severe	274	32.84	30.36	35.34	
	Total	352	30.90	28.70	33.11	
(PLATELET)	Moderate	78	294.77	268.91	320.63	.024
	Severe	274	263.05	250.24	275.86	
	Total	352	270.08	258.56	281.60	
(WBC)	Moderate	78	10.46	9.68	11.24	.398
	Severe	274	10.97	10.38	11.58	
	Total	352	10.86	10.36	11.36	
(MCV)	Moderate	78	80.82	78.95	82.70	.057
	Severe	274	83.28	82.04	84.53	
	Total	352	82.74	81.68	83.80	
(MCHB)	Moderate	78	27.82	26.91	28.73	.000
	Severe	274	30.01	29.58	30.45	
	Total	352	29.52	29.12	29.93	
(MCHBC)	Moderate	78	31.76	30.92	32.60	.827
	Severe	274	31.66	31.25	32.08	
	Total	352	31.68	31.31	32.05	

BNP = brain natriuretic peptide, CRP = C-creative protein, ESR = erythrocyte sedimentation rate, INR = international normalized ratio, LDH = lactate dehydrogenase, MCH = mean corpuscular hemoglobin, NLR = neutrophil to lymphocyte ratio, PTT = partial thromboplastin time, WBC = white blood cells.

## 4. Discussion

Our study aimed at examining the role of inflammation in determining the severity of COVID-19 among hospitalized patients with diabetes. So, various statistical analyses were done to assess the associations between the study variables; more specifically, to assess whether diabetes played a role in determining the type and strength of the relationships between inflammation and the severity of COVID-19 infection.

Our results showed that there was significant difference in term of COVID-19 severity between patient with and patients without diabetes. This finding is congruent with the literature were most of the studies that addressed this issue concluded the same finding.^[16,19]^ These findings also support the current evidence regarding the increased mortality in COVID-19 among patients with diabetes. For example, Harbuwono et al^[20]^ found that COVID-19 mortality among his study sample was 2 folds higher in patients with diabetes compared to those with no diabetes even after accounting for other comorbid conditions and accounting for diabetes complications. To explain the high mortality, several studies pointed out the possible role of inflammation specially that diabetes in known to affect the immune system. For example, Guo et al,^[21]^ indicated that dysregulated innate immune response is considered an important factor in explaining COVID-19 mortality because inflammation plays a major role in disease severity; however, these relationships are complex. This complexity was proposed because contradictory results were reported regarding the levels of the inflammatory markers and because the contradictory findings regarding the efficiency of cytokine inhibitors. In another study, study investigating the role of D-dimer test in identifying the severity of COVID-19 pandemic, Yao et al^[22]^ utilized a retrospective study design to analyze characteristics of COVID-19 illness among 248 patients.

In our study, the authors found that, among many other studied factors, the increase of D-dimer was a sole variable to increase the odds of mortality among COVID-19 patients. Thrombosis, pulmonary embolism, and deep venous thrombosis were associated with the mortality of patients. Our study concluded that D-dimer is a reliable biomarker for COVID-19 mortality and is correlated with disease severity. Similarly, In a literature review study that intended to identify the Biomarkers associated with COVID-19 disease progress, Ponti et al^[23]^ found that a multiplicity of biomarkers are associated with the disease including: (1) hematological (lymphocyte count); (2) neutrophil count; (3) NLR; (4) inflammatory (CRP); (5) ESR; (6) PCT; (7) immunological (interleukin [IL]-6) and biochemical (D-dimer, troponin, creatine kinase); and (8) aspartate aminotransferase, especially those related to coagulation cascades in disseminated intravascular coagulation and acute respiratory distress syndrome. In previous study higher levels of D-dimer were associated with worse prognosis of COVID-19 patients.^[24]^ Increase of D-dimer and fibrinogen levels by 3 or 4 times during early stages of COVID-19 was associated with higher severity. Previous studies concluded that D-dimer test is a reliable predictor of severity in COVID-19 patients and disease prognosis.^[17,24]^ Also, another study concluded that D-dimer is a reliable biomarker for COVID-19 mortality, and is correlated with disease severity.^[22]^

Our study showed that various inflammatory markers significantly influenced the severity of COVID-19 infection. Our results showed that the mean scores of neutrophil counts, monocyte count, Basophil count, ESR, PTT, CRP, platelets, white blood cells, and MCHC differed significantly between those with and those without diabetes However, the inflammatory markers that significantly affected the severity of COVID-19 among patients with diabetes were not the same ones that affected the severity in patients without diabetes. In our study, the only factor that consistently affected the severity was age. This further adds to the complexity of the topic as multiple variables seem to modulate the role of inflammation on the severity of COVID-19 and the associated health outcomes such as the level of mortality. These findings are not dissimilar from the literature as age was consistently addressed as a major influencing factor whereas the role of the inflammation was debatable.^[16,21]^

In our study, interrelations that were emerging through the results of the statistical tests between the inflammatory markers and diabetes, and the inflammatory markers and the severity of COVID-19 were multifaceted. Among the various markers that were tested, only ESR, PTT, and CRP had mean scores that significantly differed between patients with diabetes and patients with no diabetes; and patients with severe and patients with moderate COVID-19 infection. This finding highlights the role these markers and their corresponding inflammatory processes play in determining the severity of COVID-19 infection, and the potential effect they exert in determining the health outcomes of patients with COVID-19-19. Our findings regarding these interrelations are congruent with the findings in the literature^[25–27]^ who reported in their review papers that inflammation and hypercoagulation states are observed and correlated to both diabetes and severe COVID-19. However, the etiopathogenetic mechanisms underlying these relationships are not very clear.

## 5. Limitations

The findings of our study should be considered within the context of its sample and methodology. The participants were recruited using a non-probability sampling technique; a convenience sampling method, which inherently contains limitations regarding the validity of the findings and its generalizability. Also, the data was collected from the participants at a single data point. These techniques may produce biased data, and thus may limit the representation of the studies population. Along the same line, the inflammatory markers were only measured during participants’ hospitalization without performing follow-up data collection or account for the psychological factors (e.g., anxiety) that may influence the inflammation. These limitations could limit the generalizability of the findings. Therefore, the authors recommend conducting future longitudinal studies with data collection from randomly selected participants to overcome these limitations.

## 6. Conclusion

In conclusion, our study addressed the influence of having diabetes among hospitalized patients with moderate and severe COVID-19 infection. The results showed that severity of COVID-19 infection was affected by diabetes where those with diabetes had more tendency to suffer from the severe form of the disease rather that the moderate level. The severity of the disease may have been affected by the inflammatory process in the study sample. Some of the inflammatory markers were not significantly different in their levels, while others were significantly different between patients with moderate and patients with severe COVID-19 infection.

## Author contributions

**Conceptualization:** Besher A. Gharaibeh, Sawsan Abuhammad.

**Data curation:** Besher A. Gharaibeh, Sawsan Abuhammad, Obieda Haneyah.

**Formal analysis:** Besher A. Gharaibeh, Amat Alkhaleq Mehrass.

**Funding acquisition:** Besher A. Gharaibeh, Sawsan Abuhammad, Obieda Haneyah.

**Investigation:** Besher A. Gharaibeh, Obieda Haneyah, Amat Alkhaleq Mehrass.

**Methodology:** Amat Alkhaleq Mehrass.

**Project administration:** Sawsan Abuhammad.

**Resources:** Obieda Haneyah.

**Software:** Obieda Haneyah, Amat Alkhaleq Mehrass.

**Supervision:** Besher A. Gharaibeh, Obieda Haneyah, Amat Alkhaleq Mehrass.

**Validation:** Sawsan Abuhammad, Obieda Haneyah.

**Visualization:** Besher A. Gharaibeh, Sawsan Abuhammad, Amat Alkhaleq Mehrass.

**Writing – original draft:** Besher A. Gharaibeh, Sawsan Abuhammad, Amat Alkhaleq Mehrass.

**Writing – review & editing:** Sawsan Abuhammad.
